# Depolarization-Stimulated Contractility of Gastrointestinal Smooth Muscle in Calcium-Free Solution: A Review

**DOI:** 10.5402/2011/692528

**Published:** 2010-11-21

**Authors:** Emily D. Evans, Allen W. Mangel

**Affiliations:** RTI-Health Solutions, 3040 Cornwallis Drive, Research Triangle Park, NC 27709, USA

## Abstract

The membrane of most gastrointestinal smooth muscles shows slow waves, slow rhythmic changes in membrane potential. Slow waves serve to bring the membrane potential of smooth muscle cells to a threshold level that elicits a second electrical event known as the spike or action potential. The inward current of the spike, in most gastrointestinal smooth muscle preparations, is carried, at least in part, by calcium. Indeed, considering the narrow diameter of smooth muscle cells, some have hypothesized that the influx of calcium during the spike is sufficient for activation of the contractile machinery. Findings consistent with this include marked reduction in contractility during exposure of muscle segments to blockers of L-type calcium channels or following reductions in external calcium levels. However, it has also been observed that following exposure of muscle segments to external bathing solutions containing no added calcium plus 5 mM EGTA to remove any remaining extracellular calcium, contractions can be triggered following membrane depolarization. It is noteworthy that in isolated smooth muscle cells or in small muscle segments, during incubation in calcium-free solution, depolarization does not induce contractions. The present paper discusses the evidence in support of depolarization-mediated contractions occurring in gastrointestinal smooth muscle segments during incubation in solutions devoid of calcium.

## 1. Introduction

A rise in intracellular calcium is the trigger for gastrointestinal smooth muscle contractions. Under normal conditions, membrane depolarization triggers an influx of calcium and this calcium serves as an activator source for contraction. A number of studies have found that gastrointestinal smooth muscles do not show spontaneous contractile activity during exposure to calcium-free solution. In cat gastric or rat ileal muscle strips, no spontaneous contractile activity was reported in calcium-free solution containing 1 mM EGTA [1, 2]. In rat ileal tissue, neither depolarization nor acetylcholine was able to induce a contraction during exposure to calcium-free solution with 1 mM EGTA [2]. Similar effects were found in Bufo gastric smooth muscle strips [3], rat colonic muscle strips [4], and guinea pig ileal longitudinal muscle strips [5]. 

 

Thus, it has generally been accepted that a stimulated influx of calcium was required for depolarization mediated activation of contraction in gastrointestinal smooth muscle. Herein, we review evidence for the occurrence of depolarization-mediated contractility in gastrointestinal smooth muscle during incubation in calcium free solution.

## 2. Electrical Activity in Calcium-Free Solutions

**Figure 1:**
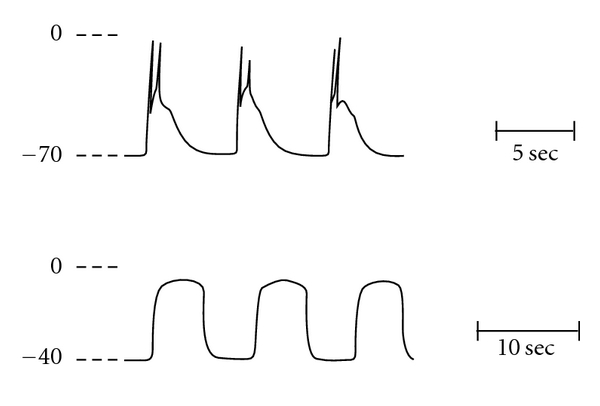
Voltage profile of electrical activity in cat small intestinal smooth muscle. Shown in the upper panel are spontaneous slow waves and spikes recorded with an intracellular microelectrode. Three slow waves are shown with spikes triggered by the depolarization associated with the upstroke of the slow wave. In the lower panel, prolonged potentials are shown following incubation in calcium-free solution. Membrane potential depolarizes from approximately −70 mV to −40 mV and the voltage excursion of the prolonged potential approaches 0 mV. Note change in time scale between the traces. From reference [9].

Electrical recordings from the plasma membrane of gastric, small intestinal, and colonic smooth muscle show slow rhythmic membrane potential changes (slow waves) that bring the membrane potential to a threshold level that triggers spikes or action potentials (Figure 1) [6, 7]. Action potentials or spikes have a voltage-dependent calcium current supporting, at least, part of their inward current. As smooth muscle cells are narrow, spindle-shaped cells, it has been suggested that the stimulated influx of calcium is sufficient to serve as activator calcium for the contractile machinery since diffusion distances from the plasma membrane to the contractile machinery would be minimized by this geometry.

Prosser et al. [8] observed in several visceral smooth muscles that following incubation of muscle segments in solution devoid of calcium and containing the calcium chelator ethylene glycol tetraacetic acid (EGTA), normal slow waves and spikes disappear and after a delay they are replaced by prolonged potentials, very slow rhythmic oscillation in membrane potential (Figure 1). Perhaps, prolonged potentials had not been observed in previous studies as a sufficient time-lag was not employed following removal of extracellular calcium. 

In calcium-free solution containing 5 mM EGTA (calcium-free hereafter), the smooth muscle resting potential depolarizes from −70 mV to approximately –40 mV, and the voltage excursion of the prolonged potential, −40 mV to 0 mV, is nearly identical to that of normal spiking [7–9] (Figure 1). The ionic basis for the prolonged potentials appears to be traversing of sodium ions through L-type calcium channels [8–10]. These events are abolished by L-type calcium channel blockers or by reduction in extracellular sodium levels.

## 3. Mechanical Activity in Calcium-Free Solution

**Figure 2:**
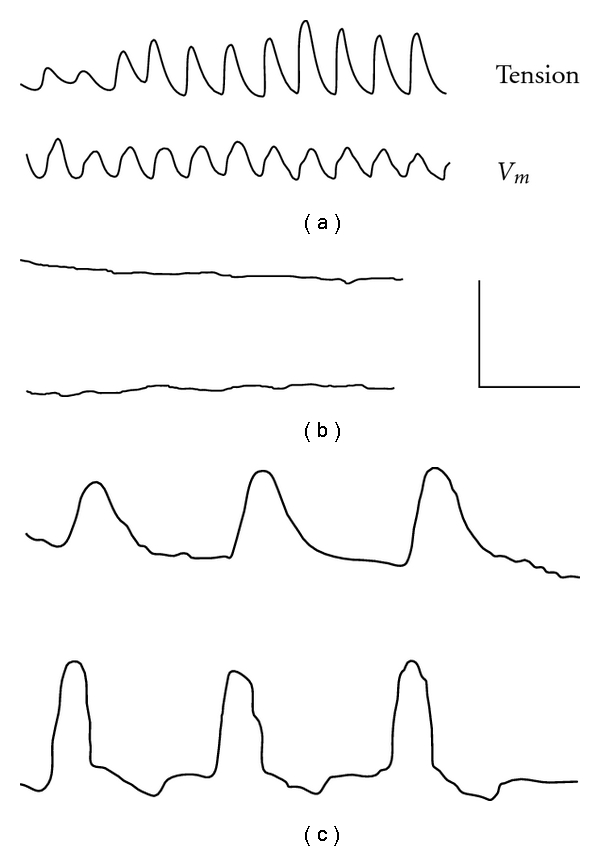
Pressure electrode recording of mechanical (upper) and electrical activity (lower traces) from a segment of cat small intestinal muscle in normal Krebs saline (a), after 7 minutes in calcium-free solution (b), and after 50 minutes in calcium-free solution (c). Following 50-minutes incubation in calcium-free solution, prolonged potentials (trace C) and corresponding contractions were observed. Calibration bar: A/B 0.4 mV, 0.8 g, 16 sec; C 0.27 mV, 0.13 g, 10 sec. From [9].

**Figure 3:**
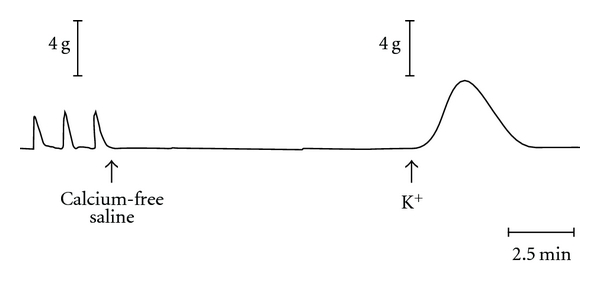
At the first arrow, the normal saline bathing this cat colonic muscle segment was removed from the bathing chamber and replaced with calcium-free solution. At the second arrow, the preparation was exposed to high-potassium (K+), calcium-free solution. From [11].

Associated with rhythmic prolonged potentials are phasic contractions that could persist for hours (Figure 2). This mechanical activity was triggered by the depolarizing phase of the prolonged potentials, suggesting the presence of an intracellular calcium store that is released during depolarization [9–13]. In preparations not showing prolonged potentials, depolarization of the plasma membrane by other means such as electrical stimulation or incubation in solutions containing high potassium levels (Figure 3) also induced mechanical activity. 

Exposure of colonic muscle segments to high potassium, calcium-free solutions showed large amplitude contractions (Figure 3) [11]. If segments were then incubated in calcium-free solutions containing normal potassium levels and then rechallenged with high potassium solution, a contraction of significantly diminished amplitude occurred. These results suggest that intracellular pools or stores are able to resequester released calcium when pulsatile release of calcium occurs during prolonged potentials; however, following large pool release as occurs with potassium depolarization, the cellular machinery is not able to resequester most of the released calcium. Thus, the resequestration machinery becomes saturated as sizeable amounts of calcium are released.

## 4. Geometry and Single Cells

Why prolonged potentials, and corresponding mechanical activity were not previously observed remains a topic for conjecture. As suggested previously, perhaps incubation times in calcium-free solutions were inadequate. An additional factor may be related to the geometry of muscle segments used. In contrast to muscle segments, evaluation of smaller muscle strips failed to demonstrate prolonged potentials and correspondingly spontaneous contractile activity was not observed [11]. This finding may be viewed as consistent with lack of depolarization-mediated stimulation of contractions in single isolated gastrointestinal smooth muscle cells or smaller muscle strips during incubation in calcium-free solution [14, 15]. Why there is a size requirement for the cellular machinery to convert to produce prolonged potentials during incubation in calcium-free solutions is not understood.

Over the past 3 decades tremendous advances in understanding the cell biology and electrophysiology of gastrointestinal smooth muscle have been gained from studies with isolated smooth muscle cells. However, we must also be sure to bear in mind that the motor functions of the gut are integrated responses and represented here is one example where translation from a cellular level to the tissue level does not occur.

## 5. Conclusions

It is clear that in a calcium-free environment, several gastrointestinal smooth muscle preparations are capable of generating an electrical event different from normal slow waves and spikes, and seen in association with these rhythmic prolonged potentials is rhythmic mechanical activity. This observation indicates that depolarization-mediated release of intracellular calcium occurs in gastrointestinal smooth muscle. Still unknown is a plausible explanation why this activity is not seen either at the single cell level or within small muscle segments. These observations provide a message about the excitation-contraction coupling process, which however, we are still not able to decipher.
